# Epidemiological characterization of congenital heart disease at different altitudes in Ecuador: a four-year retrospective study in a pediatric referral hospital

**DOI:** 10.3389/fpubh.2025.1497253

**Published:** 2025-01-29

**Authors:** Juan S. Izquierdo-Condoy, Fabian D. Arias-Rodríguez, Walter I. Díaz-Chamba, Diego A. Mena-Noroña, Lizbeth Cueva Toaquiza, Beanjuly Espín-Sambache, Emilia J. Valdivieso-Andrade, Amanda Cangas-Isacaz, Susana García-Cañarte, Mario Rubio-Niera, Esteban Ortiz-Prado

**Affiliations:** ^1^One Health Research Group, Universidad de las Américas, Quito, Ecuador; ^2^Hospital Pediátrico Baca Ortiz, Quito, Ecuador; ^3^Facultad de Ciencias Médicas, Universidad de Buenos Aires, Buenos Aires, Argentina

**Keywords:** congenital heart disease, pediatric populations, clinical records, high altitude, acyanotic, cyanotic

## Abstract

**Background:**

Congenital heart disease (CHD) is one of the main causes of hospital admissions and infant mortality, especially in developing regions.

**Objectives:**

This study aims to describe the epidemiological and clinical features of CHD in pediatric patients, from one of the largest tertiary-level national referral pediatric hospitals in Ecuador.

**Materials and methods:**

An epidemiological, retrospective cohort study was conducted among patients with CHD who were hospitalized at “Hospital Baca Ortiz” between January 2019 to December 2022 in Quito, Ecuador.

**Results:**

A total of 1,000 pediatric patient medical records were reviewed from 2019 to 2022. Among these patients, 56.0% were female, and 65.2% resided at high altitudes (2,500–3,500 m). Most cases (71.4%) were acyanotic CHD, with patent ductus arteriosus being the most prevalent (48.7%). Among cyanotic CHD, Tetralogy of Fallot was predominant (28.1%). Mortality was observed in 5.3% of patients, with a higher rate among acyanotic CHD (*p* < 0.001).

**Conclusion:**

Most cases of CHD were of the acyanotic type, predominantly affecting female patients. Although most cases occurred in children living above 2,500 meters, no clear influence of altitude on specific types of CHD was found. While mortality related to CHD was low, the burden of disability from these conditions was significant among patients with acyanotic CHD.

## 1 Introduction

Congenital heart diseases (CHD) are one of the leading causes of hospitalization and death among newborn children worldwide (1, 2). CHDs are considered the most frequent congenital anomalies, and their prevalence has been progressively increasing during this century, with a worldwide prevalence of approximately 9 to 9.4 cases per 1,000 births (3). The physio-pathological conditions are vast and depend on gross structural abnormalities of the heart or the intrathoracic great vessels (4). The exact mechanisms of CHD have not been fully elucidated, and its occurrence is multifactorial. Several factors have been recognized to increase the risk of its occurrence, including maternal factors such as excessive alcohol consumption during pregnancy, the use of medications, maternal viral infections such as rubella virus and measles in the first trimester of pregnancy (5–7).

The spectrum of CHD encompasses a diversity of anatomical and functional alterations, ranging from mild anomalies to complex malformations that require early diagnosis and medical intervention (8). It is estimated that 75% of these infants will require highly complex surgery in the first 2 years of life, and 25% will die before the first month of life. Underscoring the importance of understanding the epidemiology of these diseases to improve medical care and develop more effective prevention strategies (9).

Worldwide, patients with congenital heart disease (CHD) face complex challenges regarding diagnosis and management. Studies are scarce, and the information is not standardized. The burden of CHD is higher among developing countries, with Latin America being one of the most affected regions globally. The most updated epidemiological studies revealed a prevalence that ranges from 5 and to 12 cases per 1,000 live births (10). Estimates of the global burden of disease place South America with high mortality rates for congenital heart disease ranging from 68.5 to 113.6 per 100,000 infants in countries such as Chile and Argentina, to 113.6 to 151.9 per 100,000 infants in countries such as Ecuador and Bolivia (1).

In Ecuador, according to estimates based on data from the National Institute of Statistics and Census (INEC), approximately 2,500 children are born with CHD each year (11). While the findings of the only study performed from an ecological analysis have exposed mean prevalence rates of 70.6 cases per 10,000 live births (12), despite its importance and the significant burden of morbidity that it represents, the panorama of congenital heart disease clinical and epidemiological characteristics has not been explored in depth in Ecuador.

In this context, this research aims to describe congenital heart diseases and their related demographic, and clinical characteristics based on the exploration of clinical history records from a national tertiary-level reference pediatric hospital located in Quito, Ecuador, from 2019 to 2022.

## 2 Materials and methods

### 2.1 Study design

An epidemiological, retrospective cohort study was conducted among patients CHD who were hospitalized at “Hospital Baca Ortiz,” a specialized tertiary-level pediatric hospital in Quito, Ecuador.

### 2.2 Setting and population

Ecuador, with an area of more than 283,000 km^2^, is the smallest country in the Andean mountainous region of South America. The country is divided into four geographical regions: the coast, the highlands, the Amazon region, and the Galapagos Islands. The Ecuadorian National Health System comprises three axes: social security, the private sector (self-financed and for-profit institutions), and the public sector (free and open-access healthcare) (13). Consequently, a significant proportion of the Ecuadorian population receives care in public sector health centers.

For this research, we reviewed the medical records of pediatric patients diagnosed with congenital heart disease and treated at the Baca Ortiz Pediatric Hospital. This hospital, belonging to the public sector, located in Quito, the capital of Ecuador, at an altitude of approximately 2,850 meters above sea level (14).

The analyses for this research included records of pediatric patients attended from January 2019 to December 2022.

### 2.3 Sample

Data were collected through non-probabilistic, convenience sampling, including all consecutive medical records until at least 1,000 records were included. The sample comprised patients attended at the Baca Ortiz Pediatric Hospital during the specified period who met the selection criteria.

### 2.4 Inclusion and exclusion criteria

Inclusion criteria were based on the medical records of pediatric patients diagnosed with congenital heart disease (CHD) using all relevant International Classification of Diseases, Tenth Revision (ICD-10) Q24 codes. Eligible patients were between 0 and 17 years 11 months of age, diagnosed between January 2019 and December 2022, and treated at the Baca Ortiz Pediatric Hospital in Quito. Exclusion criteria included patients diagnosed with congenital heart conditions outside the study period or those older than 17 years 11 months, which is the maximum age for patient management and treatment at the hospital.

### 2.5 Sample and data collection

Following approval from the Institutional Review Board, we accessed the anonymized database of the Baca Ortiz Pediatric Hospital. This dataset comprised 1,049 clinical records of patients newly diagnosed with CHD between January 2019 and December 2022. The cohort included patients of advanced age referred from lower-complexity healthcare centers across Ecuador, who ultimately received their diagnoses at the Baca Ortiz Pediatric Hospital. After applying the predefined inclusion and exclusion criteria, 49 records were excluded, yielding a final dataset of 1,000 valid medical records.

From this dataset, information on demographic variables, including sex, age, ethnicity, province, and altitude of residence, which was categorized according to the altitude at which each patient’s city of residence is located based on the classification recommended by the International Society of Mountain Medicine (ISMM) which classifies altitude in 4 categories (low altitude 0–1,500 m; moderate altitude 1,500–2,500 m; high altitude 2,500–3,500 m; very high altitude 3,500 m) (15). To provide a more comprehensive understanding of this altitude variable, the average altitude of each Ecuadorian province was also determined.

Pathology analysis was based on the variable type of congenital heart disease, and additional variable data were collected to profile the characteristics of pathology management and prognosis, such as length of hospital stay and hospital discharge status.

### 2.6 Statistical analysis

Categorical variables were described using frequencies and percentages, while quantitative variables were analyzed using mean and standard deviation (SD). Chi-Square tests were employed to examine associations between categorical variables and the type of congenital heart disease. Additionally, a one-way ANOVA test was used to analyze differences between quantitative variables and the type of congenital heart disease. A *p*-value of <0.05 was considered statistically significant.

All data analyses were executed using IBM SPSS Statistics for Windows, Version 29.0 (IBM Company, Chicago, IL, United States).

## 3 Results

### 3.1 Demographic characteristics

A total of 1,000 clinical records of pediatric patients with congenital heart disease were included. Female sex predominated at 56.0% (*n* = 560), as well as mestizo ethnicity at 98.5% (*n* = 985). Likewise, the predominant age of care was between 2 and 5 years, accounting for 31% (*n* = 310) (Table 1).

**Table 1:** Demographic characteristics of patients with CHD between 2019 and 2022.

Characteristics		*n*	%
Year of attention	2019	348	34.8
	2020	204	20.4
	2021	230	23.0
	2022	218	21.8
Sex	Male	440	44.0
	Female	560	56.0
Age	0–31 days	154	15.4
	1 month—1 year	197	19.7
	2–5 years	310	31.0
	5–12 years	236	23.6
	12–18 years	103	10.3
Ethnicity	Indigenous	2	0.2
	Mestizo	985	98.5
	Other	13	1.3
Altitude of residence	Low altitude (0–1,500 m)	252	25.2
	Moderate altitude (1500–2,500 m)	96	9.6
	High altitude (2500–3,500 m)	652	65.2

**Figure 1:**
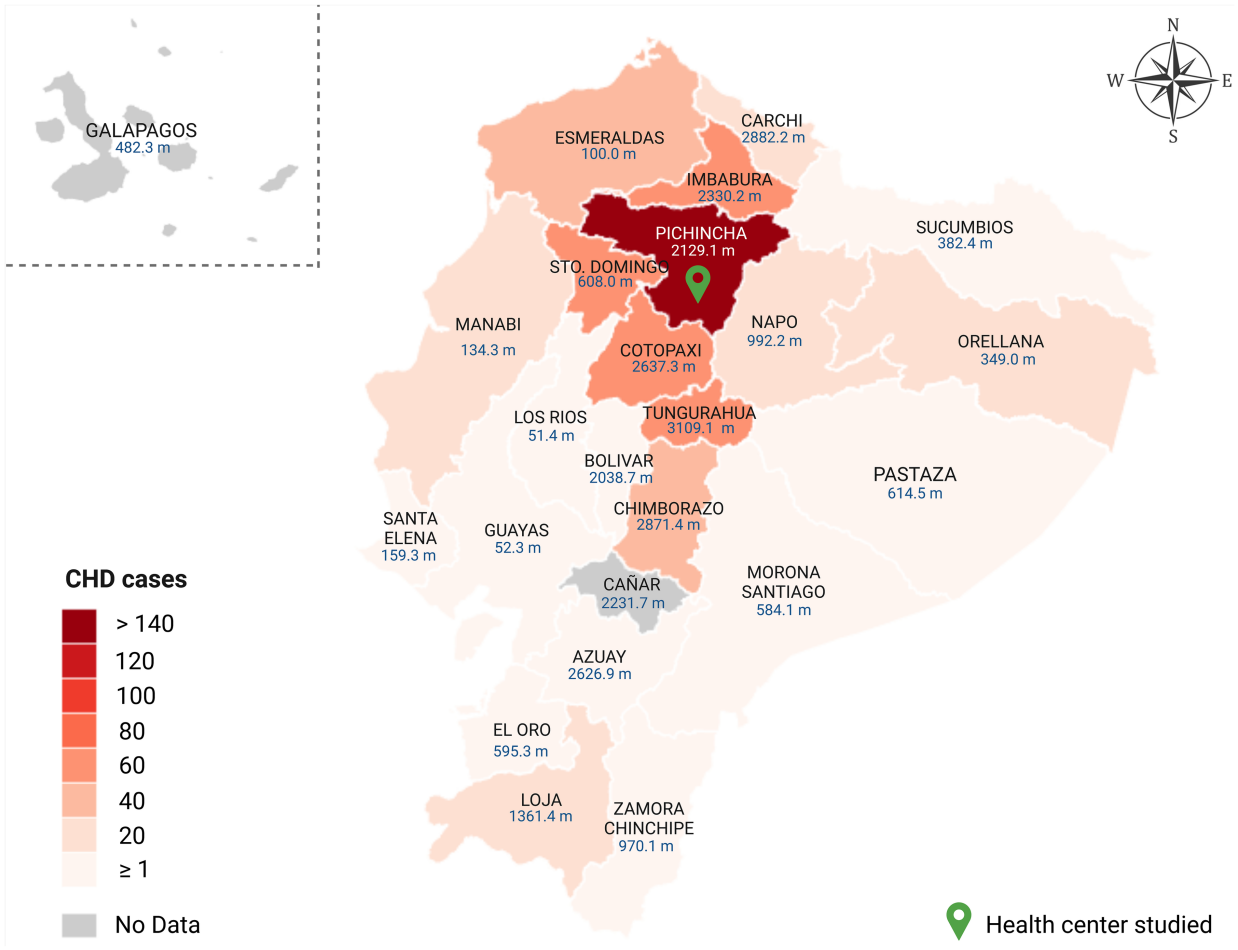
Geographical distribution of CHD cases (*n* = 1,000) according by Ecuadorian provinces between 2019 and 2022. The figure depicts the geographic origin of congenital heart disease cases included in this study, with a green pin highlighting Pichincha province, where the hospital conducting the study is located. Additionally, the map displays the average altitude of each Ecuadorian province in meters above sea level.

Regarding the geographical distribution of patients’ residence, the province with the highest number of cases was Pichincha at 47.3%, followed by Tungurahua at 7.4%, also within the Andean region. In the Coastal region, the province of Santo Domingo de los Tsachilas had the highest percentage of cases at 5.4% (Figure 1). Additionally, 65.2% (*n* = 652) of patients with congenital heart disease were found to reside at high altitudes (between 2,500 and 3,500 m) (Table 1).

### 3.2 Characteristics of congenital heart disease

The predominant group of patients had acyanotic congenital heart disease at 71.4% (*n* = 714), while the least representative group was other CHD at 5.2% (*n* = 52). Complementarity, in the analysis by the type of heart disease, in the group of acyanotic congenital heart disease, the predominant pathology was patent ductus arteriosus at 48.7% (*n* = 348), followed by atrial septal defect (26.2%). On the other hand, Tetralogy of Fallot was the most representative pathology among cyanotic CHD at 28.1% (*n* = 48). And coarctation of the aorta was the most representative CHD 49.2% (*n* = 31) within the left/right cardiac obstruction heart diseases (Table 2).

**Table 2:** Type and diagnosis of CHD in Ecuadorian pediatric patients (2019–2022).

		*n*	%
Type of congenital heart disease	Acyanotic	714	71.4
	Cyanotic	171	17.1
	Left/right heart obstructive	63	6.3
	Other	52	5.2
Diagnosis			
Acyanotic	Patent Ductus Arteriosus	348	48.7%
	Atrial Septal Defect	187	26.2%
	Atrioventricular Septal Defect	19	2.7%
	Ventricular Septal Defect	139	19.5%
	Dextrocardia	4	0.6%
	Double-entry ventricle	17	2.4%
Cyanotic	Ebstein’s Anomaly	18	10.5%
	Pulmonary Artery Atresia	26	15.2%
	Pulmonary Valve Atresia	23	13.5%
	Total Anomalous Pulmonary Venous Connection	14	8.2%
	Ventriculoarterial Connection Discordance	17	9.9%
	Hypoplastic Right Heart Syndrome	1	0.6%
	Hypoplastic Left Heart Syndrome	7	4.1%
	Tetralogy of Fallot	48	28.1%
	Transposition of the Great Vessels	14	8.2%
Left/right heart obstructive	Aortic Atresia	11	17.5%
	Coarctation of the Aorta	31	49.2%
	Congenital Stenosis of the Aortic Valve	4	6.3%
	Congenital Stenosis of the Pulmonary Valve	10	15.9%
	Pulmonary Artery Stenosis	2	3.2%
	Congenital Subaortic Stenosis	5	7.9%
Other	Partial Anomalous Pulmonary Venous Connection	6	11.5%
	Triatrial Heart	2	3.8%
	Congenital Tricuspid Valve Stenosis	16	26.9%
	Congenital Mitral Stenosis	1	1.9%
	Congenital Aortic Valve Insufficiency	1	1.9%
	Congenital Mitral Insufficiency	1	1.9%
	Congenital Malformation of the Tricuspid Valve, Not Specified	3	5.8%
	Congenital Malformation of the Heart, Unspecified	2	3.8%
	Congenital Malformation of Coronary Vessels	3	3.8%
	Other Congenital Malformations of the Aorta	5	9.6%
	Other Congenital Malformations of the Pulmonary Artery	3	5.8%
	Other Congenital Malformations of the Tricuspid Valve	1	1.9%
	Other Congenital Malformations of the Cardiac Chambers and Their Connections	2	3.8%
	Other Congenital Malformations of the Great Arteries	1	1.9%
	Other Congenital Malformations of the Great Veins	4	7.7%
	Other Congenital Malformations of the Heart, Specified	2	3.8%
	Common Truncus Arteriosus	2	3.8%
Outcome			
Length of stay (days), Mean (± SD)		8.4	± 15.5
Discharge condition	Discharged	927	92.7
	Disability	20	2.0
	Deceased	53	5.3

Concerning hospital management, the mean hospital stay for patients with CHD was 8.4 days with a standard deviation of 15.5. In terms of discharge condition, 5.3% (*n* = 53) of patients died, and 2.0% (*n* = 20) had some type of disability (Table 2).

### 3.3 Differences between congenital heart disease types

When analyzing the possible influence within the type of congenital heart disease, we found that the female group was representative among acyanotic heart diseases (76.1%) (*p* = 0.002). In turn, among age groups, acyanotic pathologies were between 2 and 5 years (81.9%), while within cyanotic heart diseases, neonates (between 0 and 31 days) had the highest frequency at 35.1% (*p* < 0.001) (Table 3).

**Table 3:** Difference of distribution of type of CHD among pediatric patients’ characteristics.

		Type of congenital heart disease								
		Acyanotic		Cyanotic		Left/right heart obstructive		Other		*p* value
		*n*	%	*n*	%	*n*	%	*n*	%	
Sex	Male	288	65.5	85	19.3	36	8.2	31	7.0	0.002
	Female	426	76.1	83	14.8	27	4.8	24	4.3	
Age	0–31 days	74	48.1	54	35.1	16	10.4	10	6.5	< 0.001
	1 month—1 year	117	59.4	47	23.9	16	8.1	17	8.6	
	2–5 years	254	81.9	36	11.6	8	2.6	12	3.9	
	5–12 years	182	77.1	26	11.0	17	7.2	11	4.7	
	12–18 years	87	84.5	5	4.9	6	5.8	5	4.9	
Ethnicity	Indigenous	2	100.0	0	0.0	0	0.0	0	0.0	0.949
	Mestizo	702	71.3	166	16.9	62	6.3	55	5.6	
	Other	10	76.9	2	15.4	1	7.7	0	0.0	
Altitude of residence	Low altitude (0–1,500 m)	168	66.7	61	24.2	14	5.6	9	3.6	0.014
	Moderate altitude (1500–2,500 m)	66	68.8	17	17.7	6	6.3	7	7.3	
	High altitude (2500–3,500 m)	480	73.6	90	13.8	43	6.6	39	6.0	
Length of stay (days), *Mean (± SD)*		8.1	14.8	8.7	15.7	11.6	15.4	9.7	19.5	0.002*
Discharge condition	Discharged	686	74.0	143	15.4	51	5.5	47	5.1	< 0.001
	Disability	8	40.0	6	30.0	4	20.0	2	10.0	
	Deceased	20	37.7	19	35.8	8	15.1	6	11.3	

*p-value calculated from one-way ANOVA.

## 4 Discussion

This study provides a comprehensive analysis of CHD among pediatric patients in Ecuador, based on data from a leading referral hospital over a four-year period (2019–2022). As the first of its kind in Ecuador, this research offers valuable insights into the clinical and epidemiological characteristics of CHD in a region with significant geographic and demographic diversity. Our findings highlight trends in gender distribution, ethnic prevalence, and the potential impact of environmental factors such as altitude, contributing to a better understanding of CHD in this unique context.

Our results demonstrated a slight predominance of cases among female pediatric patients (56.0%) (ratio 1.3:1), a trend also found among children from Tibet (16). This finding is consistent with previous research on Ecuadorian adults with CHD by Robles T. and Lopez J., who reported 58.6% females (17), and Kwag et al. in a long-term analysis of adults from Korea (18). However, this predominance contrasts with findings from studies in pediatric settings globally, including Egypt, Sudan, China, Iran, Nigeria, and Colombia, where males were predominantly affected by CHD (19–25).

Regarding ethnic characteristics, previous research has shown that Hispanics have a higher incidence of congenital heart disease compared to non-Hispanic white and non-Hispanic black populations (26). In this context, our findings indicated an almost absolute predominance of CHD in the mestizo ethnicity. However, this factor cannot be considered associated with the development of CHD, given that the majority of the Ecuadorian population belongs to the mestizo ethnicity (27).

Among environmental influences, altitude has been shown to have a significant association with CHD (16, 28, 29). In this study, 65.2% of cases occurred at high altitudes (2,500–3,500 m); consequently, most cases involved patients residing in provinces located in the Sierra region of Ecuador, including Pichincha, Tungurahua, and Cotopaxi, all located above 2,500 meters. Similar distributions have been identified in Colombia (30), and in the ecological exploration by Gonzales Andrade in Ecuador using secondary data, which identified a higher prevalence at altitudes above 2,500 m (12). Despite these findings, our ability to establish a direct causal relationship between altitude and CHD is limited due to the location of the study hospital in Quito, situated at an altitude of 2,850 meters. This proximity likely influenced the sample population, as patients from high-altitude regions were more likely to seek care at this facility. However, a recent multinational study, including Ecuador and several other countries, corroborated a higher prevalence of CHD at high altitudes, reporting rates of 14.47% at 2,500–3,500 meters and 7.26% at 3,500–4,500 meters (31).

The increased occurrence of CHD at high altitudes can be attributed to both biological and socio-environmental factors. One key biological factor is chronic hypoxia during pregnancy, which is common in high-altitude regions. Hypoxia can compromise fetal oxygenation during critical periods of cardiovascular development, increasing the risk of structural heart defects (32, 33). Additionally, hypoxia reduces placental blood flow and nutrient transfer, which may lead to restricted fetal growth and abnormal blood flow patterns. These changes can increase pulmonary vascular resistance, contributing to structural anomalies such as ventricular septal defects (34–36).

Additionally, nutritional deficiencies common in high-altitude regions may significantly contribute to the elevated risk of CHD. Limited access to diverse and nutrient-rich food sources has been associated with deficiencies in critical nutrients such as folic acid, a key component in cardiac development (37, 38). Furthermore, socioeconomic barriers, such as inadequate access to healthcare services and prenatal care, may exacerbate these risks. In high-altitude areas, restricted healthcare access can delay early diagnosis and management, potentially increasing CHD incidence (34). These challenges have been similarly observed in Ecuador, where limited healthcare resources at high altitudes are well-documented (39).

Regarding the clinical classification of congenital heart disease, we found a marked predominance of acyanotic congenital heart disease (71.4%). These findings are consistent with studies exploring pediatric populations in Jordan (60.1%), Iran (77.2%), Nigeria (83.4%), Egypt (79.2%), and the Dominican Republic (82.9%) (19, 22, 23, 28, 40). Furthermore, the most frequent acyanotic congenital heart disease identified was patent ductus arteriosus (45.8% among acyanotic heart diseases and 34.8% of CHDs overall), followed by atrial septal defect (26.2%) and ventricular septal defect (18.3%). This trend has been observed in other examinations, including pediatric patients from Tibet and Colombia (16, 30).

On the other hand, among cyanotic congenital heart diseases, tetralogy of Fallot was the most frequent (46.6%), followed by Ebstein’s anomaly (17.5%). Tetralogy of Fallot has previously been identified as the most frequent in Egyptian, Sudanese, and Cuban children (19, 20, 41).

According to the classification of CHD types, we observed a possible association between female sex, patients between 2 and 5 years old, high altitude, and acyanotic cardiopathies. This may be due to the predominance of these three groups in this study. Additionally, several investigations have shown a high prevalence of acyanotic congenital heart disease in high-altitude regions such as Tibet, the mountainous areas of India, and the Himalayas (16, 28, 42). In this context, we consider that studies with robust methodologies and representative samples are needed to elucidate the true effect of altitude on CHD and its types.

Regarding the prognosis of patients with CHD, mortality attributable to congenital heart disease in this study was 5.3% of the total sample, lower than that found by do Amaral et al. in Brazilian pediatric patients (12.0%) (43). Among our patients, the majority of deaths occurred in those with acyanotic congenital heart disease. On the other hand, although only 2.0% of cases resulted in disability, there was evidence of an association between this outcome and the type of acyanotic CHD, which includes patent ductus arteriosus and atrial septal defect, among others. The main types leading to mortality were acyanotic and cyanotic CHD, showing a significant burden for these types of CHD.

CHD poses a substantial public health burden in Ecuador and other low- and middle-income countries (LMICs), impacting not only healthcare systems but also the lives of patients and their families. Rapid diagnosis and timely treatment are crucial in mitigating the physical and psychological effects of these conditions. In the context of LMICs, where healthcare access can be limited, improving healthcare strategies and policies is essential to enhance outcomes for children with CHD. This underscores the importance of targeted interventions and the need for comprehensive healthcare planning to address the unique challenges faced by pediatric patients with congenital heart disease in these regions.

### 4.1 Limitations

This research encountered several limitations. The reliance on data from a single tertiary-level pediatric hospital in Quito, situated at an altitude of 2,850 meters, likely introduced selection bias, resulting in an overrepresentation of patients from high-altitude regions and limiting the generalizability of findings to the wider Ecuadorian population. As a retrospective cohort study, the research was dependent on existing medical records, which were susceptible to inconsistencies and incomplete data, particularly in diagnostic and demographic variables, thereby constraining the verification of accuracy. The study design did not account for critical confounding factors, including maternal health, the quality of prenatal care, and genetic predispositions, all of which could significantly influence the incidence and outcomes of CHD. Additionally, socioeconomic and regional disparities may have biased the sample, underrepresenting CHD cases from rural or underserved areas. While data collection involved a multidisciplinary team, this approach raised the risk of inconsistencies, though rigorous review processes were implemented to minimize errors. Despite these efforts, the findings may not comprehensively reflect the epidemiology of CHD across Ecuador. Future research should aim to overcome these limitations by adopting multicenter designs, prospective methodologies, and a more extensive range of variables to provide a more representative and nuanced understanding of CHD in diverse populations.

## 5 Conclusion

This study found a predominance of CHD among female patients, particularly acyanotic CHD, with patent ductus arteriosus being the most common. Tetralogy of Fallot was the most frequent among cyanotic CHDs. Most cases were in children living at altitudes higher than 2,500 meters, though no significant association between altitude and specific CHD types was established. Mortality due to CHD was relatively low, but the burden of disabling conditions was notable among patients with other CHDs. Further studies on CHD in high-altitude regions like Ecuador are essential to understand the impact of altitude on these conditions. Enhanced academic and health initiatives are needed to address CHDs like patent ductus arteriosus, which have significant physical and psychological effects.

## Data Availability

The original contributions presented in the study are included in the article/supplementary material, further inquiries can be directed to the corresponding author.

## References

[1] Zimmerman MS Smith AGC Sable CA Echko MM Wilner LB Olsen HE. Global, regional, and national burden of congenital heart disease, 1990–2017: a systematic analysis for the global burden of disease study 2017. Lancet Child Adolesc Health. (2020) 4:185–200. doi: 10.1016/S2352-4642(19)30402-X, PMID: 31978374 PMC7645774

[2] Hoffman JI. The global burden of congenital heart disease. Cardiovasc J Afr. (2013) 24:141–5. doi: 10.5830/CVJA-2013-028, PMID: 24217047 PMC3721933

[3] Liu Y Chen S Zühlke L Black GC Choy M-K Li N. Global birth prevalence of congenital heart defects 1970-2017: updated systematic review and meta-analysis of 260 studies. Int J Epidemiol. (2019) 48:455–63. doi: 10.1093/ije/dyz009, PMID: 30783674 PMC6469300

[4] Mitchell SC Korones SB Berendes HW. Congenital heart disease in 56,109 births. Incidence Nat His Circul. (1971) 43:323–32. doi: 10.1161/01.cir.43.3.323, PMID: 5102136

[5] Sun R Liu M Lu L Zheng Y Zhang P. Congenital heart disease: causes, diagnosis, symptoms, and treatments. Cell Biochem Biophys. (2015) 72:857–60. doi: 10.1007/s12013-015-0551-6 25638345

[6] Ogeng JA Gatonga PM Olabu BO Nyamweya DK Ong’era D. Pattern of congestive heart failure in a Kenyan paediatric population. Cardiovasc J Afr. (2013) 24:117–20. doi: 10.5830/CVJA-2013-015, PMID: 24217041 PMC3734873

[7] Roodpeyma S Kamali Z Afshar F Naraghi S. Risk factors in congenital heart disease. Clin Pediatr (Phila). (2002) 41:653–8. doi: 10.1177/000992280204100903, PMID: 12462314

[8] Zheng G Wu J Chen P Hu Y Zhang H Wang J. Characteristics of in-hospital mortality of congenital heart disease (CHD) after surgical treatment in children from 2005 to 2017: a single-center experience. BMC Pediatr. (2021) 21:521. doi: 10.1186/s12887-021-02935-2, PMID: 34814864 PMC8609813

[9] Liu Y Chen S Zühlke L Babu-Narayan SV Black GC Choy M-K. Global prevalence of congenital heart disease in school-age children: a meta-analysis and systematic review. BMC Cardiovasc Disord. (2020) 20:488. doi: 10.1186/s12872-020-01781-x, PMID: 33213369 PMC7678306

[10] Botto LD Correa A Erickson JD. Racial and temporal variations in the prevalence of heart defects. Pediatrics. (2001) 107:E32. doi: 10.1542/peds.107.3.e32, PMID: 11230613

[11] Paucar E. 2 500 niños nacen cada año con cardiopatías en Ecuador. El Comercio (2023). Available at: https://www.elcomercio.com/tendencias/salud/2-500-ninos-nacen-cada-ano-con-cardiopatias-en-ecuador.html (Accessed April 8, 2024)

[12] González-Andrade F. High altitude as a cause of congenital heart defects: a medical hypothesis rediscovered in Ecuador. High Alt Med Biol. (2020) 21:126–34. doi: 10.1089/ham.2019.0110, PMID: 31976751

[13] Instituto Nacional de Estadística y Censos. Actividades y Recursos de Salud. Instituto Nacional de Estadística y Censos INEC (2023). Available at: https://www.ecuadorencifras.gob.ec/actividades-y-recursos-de-salud/ (Accessed April 9, 2024)

[14] Ministerio de Salud Pública de Ecuador. Hospital Pediátrico “Baca Ortiz.” Hospital Pediátrico “Baca Ortiz” (2022) Available at: http://www.hbo.gob.ec/index.php/56-direccion/91-ubicacion-del-hospital (Accessed April 9, 2024)

[15] Imray C Booth A Wright A Bradwell A. Acute altitude illnesses. BMJ. (2011) 343:d4943–3. doi: 10.1136/bmj.d4943, PMID: 21844157

[16] Chun H Yue Y Wang Y Dawa Z Zhen P La Q. High prevalence of congenital heart disease at high altitudes in Tibet. Eur J Prev Cardiol. (2019) 26:756–9. doi: 10.1177/2047487318812502, PMID: 30419180

[17] Robles Lituma TVR López Rodríguez JA. Incidencia de cardiopatías congénitas en población adulta en el Hospital de Especialidades José Carrasco Arteaga—Cuenca entre 2015 y 2020: Incidence of congenital heart disease in adult population at Hospital de Especialidades José Carrasco Arteaga—Cuenca between 2015 and 2020. Rev Med HJCA. (2022) 14:102–7. doi: 10.14410/2022.14.2.ao.16

[18] Kwag EM Lee JS Kim SH. The incidentally diagnosed adult congenital heart disease during routine medical health checkups in 27,897 Koreans at a single center over seven years. BMC Cardiovasc Disord. (2018) 18:223. doi: 10.1186/s12872-018-0968-0, PMID: 30518327 PMC6280454

[19] Al-Fahham MM Ali YA. Pattern of congenital heart disease among Egyptian children: a 3-year retrospective study. Egypt Heart J. (2021) 73:11. doi: 10.1186/s43044-021-00133-0, PMID: 33512632 PMC7846646

[20] Abdelrahman O Diab R. Prevalence and pattern of congenital heart disease among children in Khartoum state, Sudan: a reflection of the current cardiac profile. Cureus. (2022) 14:e21196. doi: 10.7759/cureus.21196 35047315 PMC8756553

[21] Zhang L Liu B Li H Wang C Yang S Li Z. Epidemiology of congenital heart disease in Jinan, China from 2005 to 9: a time trend analysis. Front Cardiovasc Med. (2022):9. doi: 10.3389/fcvm.2022.815137, PMID: 35571178 PMC9092597

[22] Parvar SY Ghaderpanah R Naghshzan A. Prevalence of congenital heart disease according to the echocardiography findings in 8145 neonates, multicenter study in southern Iran. Health Sci Rep. (2023) 6:e1178. doi: 10.1002/hsr2.1178, PMID: 37033389 PMC10073012

[23] Otaigbe BE Tabansi PN. Congenital heart disease in the Niger Delta region of Nigeria: a four-year prospective echocardiographic analysis. Cardiovasc J Afr. (2014) 25:265–8. doi: 10.5830/CVJA-2014-055, PMID: 25388927 PMC4327180

[24] Tassinari S Martínez-Vernaza S Erazo-Morera N Pinzón-Arciniegas MC Gracia G Zarante I. Epidemiology of congenital heart diseases in Bogotá, Colombia, from 2001 to 2014: improved surveillance or increased prevalence? Biomedica. (2018) 38:148–55. doi: 10.7705/biomedica.v38i0.3381, PMID: 29809331

[25] Nazari P Davoodi M Faramarzi M Bahadoram M Dorestan N. Prevalence of congenital heart disease: a single center experience in southwestern of Iran. Glob J Health Sci. (2016) 8:288. doi: 10.5539/gjhs.v8n10p288, PMID: 27302457

[26] Nembhard WN Salemi JL Wang T Loscalzo ML Hauser KW. Is the prevalence of specific types of congenital heart defects different for non-Hispanic white, non-Hispanic black and Hispanic infants? Matern Child Health J. (2010) 14:184–93. doi: 10.1007/s10995-009-0442-9 19169800

[27] Instituto Nacional de Estadística y Censos INEC. País atrevido: la nueva cara sociodemográfica del Ecuador. (2013) Available at: https://web.archive.org/web/20130918214724/http://www.inec.gob.ec/publicaciones_libros/Nuevacarademograficadeecuador.pdf# (Accessed July 29, 2022)

[28] Hasan A. Relationship of high altitude and congenital heart disease. Indian Heart J. (2016) 68:9–12. doi: 10.1016/j.ihj.2015.12.015, PMID: 26896259 PMC4759507

[29] Miao C-Y Zuberbuhler JS Zuberbuhler JR. Prevalence of congenital cardiac anomalies at high altitude. J Am Coll Cardiol. (1988) 12:224–8. doi: 10.1016/0735-1097(88)90378-6, PMID: 3379209

[30] Gómez-Monroy CA Hoyos-Gómez LK Acosta-Costilla ÁF Muñoz-Torres LD Fernández-Ávila DG. Prevalence of congenital heart disease in relation to height above sea level in a region of Colombia. Arch Cardiol Mex. (2023) 93:37–43. doi: 10.24875/ACM.21000325, PMID: 36757777 PMC10161842

[31] Zila-Velasque JP Grados-Espinoza P Cubas WS Diaz-Barrera M Pacheco-Mendoza J. Analysis of congenital heart disease research: mapping impact, production and global collaboration. Heliyon. (2023) 9:e19188. doi: 10.1016/j.heliyon.2023.e19188, PMID: 37649838 PMC10462836

[32] Giussani DA Davidge ST. Developmental programming of cardiovascular disease by prenatal hypoxia. J Dev Orig Health Dis. (2013) 4:328–37. doi: 10.1017/S204017441300010X, PMID: 24970726

[33] Moore LG. Measuring high-altitude adaptation. J Appl Physiol (1985). (2017) 123:1371–85. doi: 10.1152/japplphysiol.00321.2017 28860167 PMC5792094

[34] Zamudio S. High-altitude hypoxia and preeclampsia. Front Biosci. (2007) 12:2967–77. doi: 10.2741/2286 17485273 PMC6428070

[35] Moore LG. Fetal growth restriction and maternal oxygen transport during high altitude pregnancy. High Alt Med Biol. (2003) 4:141–56. doi: 10.1089/152702903322022767, PMID: 12855048

[36] Keyes LE Armaza JF Niermeyer S Vargas E Young DA Moore LG. Intrauterine growth restriction, preeclampsia, and intrauterine mortality at high altitude in Bolivia. Pediatr Res. (2003) 54:20–5. doi: 10.1203/01.PDR.0000069846.64389.DC, PMID: 12700368

[37] Reynolds RD Marriott BM Carlson SJ. “Effects of cold and altitude on vitamin and mineral requirements.,” Nutritional needs in cold and in high-altitude environments: Applications for military personnel in field operations. Washington, D.C: National Academies Press (US) (1996). Available at: https://www.ncbi.nlm.nih.gov/books/NBK232871/ (Accessed January 6, 2025) 25121290

[38] Molloy AM Kirke PN Brody LC Scott JM Mills JL. Effects of folate and vitamin B12 deficiencies during pregnancy on fetal, infant, and child development. Food Nutr Bull. (2008) 29:S101–11. doi: 10.1177/15648265080292S114 18709885

[39] DeWit J. Healthcare in Rural Ecuador. StoryMaps (2024) Available at: https://storymaps.com/stories/298d6937b78141f5856144f61c745d4a (Accessed January 6, 2025)

[40] Mancebo García P Soriano N Lazala LE Contreras E Haché C Malagón L. Incidencia de cardiopatías congénitas en pacientes ingresados en la unidad de Neonatología del Hospital Infantil Robert Reid Cabral, 2016-2018. Ciencia y Salud. (2022) 6:43–8. doi: 10.22206/cysa.2022.v6i2.pp43-48

[41] Clemades Méndez AM Rodríguez Díaz LK Salazar Sotolongo A. Cardiopatías congénitas en el recién nacido. aniversariocimeq2021. (2021). p. 1–14. Available at: https://aniversariocimeq2021.sld.cu/index.php/ac2021/Cimeq2021/paper/view/177 (Accessed April 9, 2024)

[42] Shah GS Singh MK Pandey TR Kalakheti BK Bhandari GP. Incidence of congenital heart disease in tertiary care hospital. Kathmandu Univ Med J (KUMJ). (2008) 6:33–6. PMID: 18604112

[43] do Amaral Lopes SAV Britto Guimarães IC de Oliva Costa SF Acosta AX Sandes KA CMC M. Mortality for critical congenital heart diseases and associated risk factors in newborns. A cohort study. Arq Bras Cardiol. (2018) 111:666–73. doi: 10.5935/abc.20180175, PMID: 30281694 PMC6248247

